# Various Effects of Sandblasting of Dental Restorative Materials

**DOI:** 10.1371/journal.pone.0147077

**Published:** 2016-01-14

**Authors:** Goro Nishigawa, Yukinori Maruo, Masao Irie, Naoto Maeda, Kumiko Yoshihara, Noriyuki Nagaoka, Takuya Matsumoto, Shogo Minagi

**Affiliations:** 1 Occlusion and Removable Prosthodontics, Okayama University Hospital, Okayama, Japan; 2 Department of Biomaterials, Okayama University Graduate School of Medicine, Dentistry and Pharmaceutical Sciences, Okayama, Japan; 3 Department of Occlusal and Oral Functional Rehabilitation, Okayama University Graduate School of Medicine, Dentistry and Pharmaceutical Sciences, Okayama, Japan; 4 Center for Innovative Clinical Medicine, Okayama University Hospital, Okayama, Japan; 5 Laboratory for Electron Microscopy, Okayama University Graduate School of Medicine, Dentistry and Pharmaceutical Sciences, Okayama, Japan; Duke University Marine Laboratory, UNITED STATES

## Abstract

**Background:**

Sandblasting particles which remain on the surfaces of dental restorations are removed prior to cementation. It is probable that adhesive strength between luting material and sandblasting particle remnants might exceed that with restorative material. If that being the case, blasting particles adhere to sandblasted material surface could be instrumental to increasing adhesive strength like underlying bonding mechanism between luting material and silanized particles of tribochemical silica coating-treated surface. We hypothesize that ultrasonic cleaning of bonding surfaces, which were pretreated with sandblasting, may affect adhesive strength of a resin luting material to dental restorative materials.

**Methods:**

We therefore observed adhesive strength of resin luting material to aluminum oxide was greater than those to zirconia ceramic and cobalt-chromium alloy beforehand. To measure the shear bond strengths of resin luting material to zirconia ceramic and cobalt-chromium alloy, forty specimens of each restorative material were prepared. Bonding surfaces were polished with silicon abrasive paper and then treated with sandblasting. For each restorative material, 40 sandblasted specimens were equally divided into two groups: ultrasonic cleaning (USC) group and non-ultrasonic cleaning (NUSC) group. After resin luting material was polymerized on bonding surface, shear test was performed to evaluate effect of ultrasonic cleaning of bonding surfaces pretreated with sandblasting on bond strength.

**Results:**

For both zirconia ceramic and cobalt-chromium alloy, NUSC group showed significantly higher shear bond strength than USC group.

**Conclusions:**

Ultrasonic cleaning of dental restorations after sandblasting should be avoided to retain improved bonding between these materials.

## Introduction

The topic of bond strength between dental restorative material and luting materials has been extensively discussed. To date, sandblasting hails as one of the most effective methods of increasing bond strength between resin luting materials and dental restorative materials [1, 2]. Roughening the surface of a restorative material, with the use of abrasive particles, brings about morphological changes and an increase in adhesive strength between the luting material and restorative material. After sandblasting, abrasive particles which remain on the surfaces of dental restorations are generally considered as pollutants and are removed using an ultrasonic cleaner prior to cementation [3, 4].

Tribochemical silica coating (TBC) is another favored method of increasing the bond strength between resin luting materials and dental restorations [5, 6]. After abrading and coating the surfaces of dental restorations with silica-coated alumina particles using the TBC technique, the use of a silane coupling agent enhances the bond strength by providing chemical bonding between the treated surface and resin luting material. Manufacturer of TBC devices (3M ESPE Dental Products, Seefeld, Germany) advises against cleaning TBC-treated surfaces with water. It was reported that ultrasonic cleaning decreased the silica content of TBC-treated surface and adversely affected the latter’s adhesive strength to resin luting material [7].

After sandblasting, some abrasive particles adhere to the blasted surface. It is probable that the adhesive strength between a luting material and the abrasive particle remnants might exceed that with the restorative material. If that being the case, abrasive particles which adhere to sandblasted material surface could be instrumental to increasing adhesive strength, like the underlying bonding mechanism between a luting material and silanized particles of TBC-treated surface. Therefore, routine cleaning and removal of abrasive particle remnants from the sandblasted surfaces of dental restorations might unwittingly reduce the adhesive strength of luting materials to restorations.

Zirconia ceramics are becoming a favored choice for fixed prostheses because of their fracture toughness and esthetic appeal. However, they exhibit poor adhesive strength to resin luting materials, except with the use of special monomers such as 10-methacryloyloxydecyl dihydrogen phosphate (MDP) [8]. For many years, cobalt-chromium (Co-Cr) alloy is the material of choice for removable prosthesis frameworks. Similarly, this material exhibits poor adhesion to denture base acrylic resins except with the use of special primers.

Aluminum oxide is a popular sandblasting abrasive medium. In this paper, the authors examined if the adhesive strength of a resin luting material to aluminum oxide was greater than those to zirconia ceramic and Co-Cr alloy. This leads to the hypothesis that rinsing aluminum oxide abrasive particles off the sandblasted surfaces of zirconia ceramics and Co-Cr alloy might decrease the adhesive strength of resin luting material to these restorative materials. The authors tested this hypothesis by examining the adhesive strengths of a resin luting material to alumina-blasted zirconia ceramic and Co-Cr alloy surfaces with and without ultrasonic cleaning. If the adhesive strength without ultrasonic cleaning was higher than that with ultrasonic cleaning, it would imply that sandblasting not only brings about morphological changes of material surface, but also increased adhesion efficiency.

## Materials and Methods

### 1 Comparison of adhesive strengths of resin luting material to aluminum oxide and dental restorative materials

#### 1.1 Specimen preparation

Three materials were tested: aluminum oxide (VITA In-Ceram ALUMINA, VITA Innovation Professionals, Bad Sackingen, Germany), yttria-stabilized tetragonal zirconia polycrystal (Y-TZP) ceramic (Lava, 3M ESPE Dental Products, Seefeld, Germany), and Co-Cr alloy (Cobaltan, Shofu Co., Japan). These three materials were cut from blocks of each material. For each material, 11 slabs (approximately 6×6 mm) were prepared using a low-speed cutting machine and embedded in an epoxy resin (SpeciFix-20 Kit, Struers A/S, Rodovre, Denmark).

The bonding surface of each specimen slab was polished with #1200 silicon carbide abrasive paper (Struers A/S, Denmark) under water irrigation to remove pollutants and obtain a flat uniform surface as previously described [9, 10]. After polishing, specimens were cleaned with distilled water using an ultrasonic cleaner (USD-1R, AS ONE, Osaka, Japan) at 40 kHz for 5 minutes to remove all debris.

A resin luting material (Panavia F 2.0, Kuraray Noritake Dental, Okayama, Japan) was polymerized on the bonding surface, in the form of a cylinder, using a Teflon mold. Teflon molds were used because Teflon does not react with the luting material. Each Teflon mold (*n* = 11 per material), of 2.0 mm depth and 3.6 mm diameter, was placed on the polished and cleaned bonding surface and filled with the luting material using a syringe tip.

Luting material was polymerized using a light curing unit (New Light VL-II, GC Corp., Tokyo, Japan; light guide tip diameter: 8 mm). Before applying curing light to the resin luting material, its irradiance was checked using a radiometer (Demetron/Kerr, Danbury, CT, USA). During polymerization, light irradiance was maintained at 450 mW/cm^2^ for 30 seconds. After polymerization was completed, all specimens were stored in 37°C distilled water for 24 hours.

#### 1.2 Shear bond strength measurement and statistical analysis

Shear bond strengths were measured after 24-hour storage in distilled water. For each material, specimens were mounted on a universal testing machine (Autograph DCSC-2000, Shimadzu, Kyoto, Japan) and then shear stress was applied at a crosshead speed of 0.5 mm/minute. Data obtained for the three materials were statistically compared using one-way analysis of variance (ANOVA) and Fisher’s LSD method.

### 2 Effect of alumina particle remnants on adhesive strength between resin luting material and sandblasted surfaces of zirconia ceramic and Co-Cr alloy

#### 2.1 Specimen preparation

For both Y-TZP ceramic (Lava, 3M ESPE Dental Products, Seefeld, Germany) and Co-Cr alloy (Cobaltan, Shofu Co., Japan), 40 slabs (approximately 6×6 mm) were prepared for each material using a low-speed cutting machine and embedded in an epoxy resin. The bonding surface of each specimen slab was polished with #1200 silicon carbide abrasive paper as described in previous head. After polishing, the bonding surface was air-abraded using a laboratory sandblaster (Hi-Blaster III, Shofu Co., Kyoto, Japan; particle size: 50-μm aluminum oxide). Distance between nozzle and bonding surface was 5 mm, and sandblasting was performed at an air pressure of 0.4 MPa for 10 seconds.

After sandblasting, 40 specimens of each material were equally divided into two groups: ultrasonic cleaning (USC) group *versus* non-ultrasonic cleaning (NUSC) group. All USC specimens were cleaned with distilled water in an ultrasonic bath (USD-1R, AS ONE, Osaka, Japan) at 40 kHz for 5 minutes, and then left to air-dry for 240 minutes. For NUSC specimens, their bonding surfaces were not cleaned ultrasonically but only with a soft air blow for 5 seconds.

A resin luting material (Panavia F 2.0, Kuraray Noritake Dental, Okayama, Japan) was polymerized on the bonding surface, in the form of a cylinder, using a Teflon mold. Each Teflon mold (*n* = 20 per group), of 2.0 mm depth and 3.6 mm diameter, was placed on the treated bonding surface and filled with the luting material using a syringe tip.

Luting material was polymerized using a light curing unit. Before applying curing light to the resin luting material, its irradiance was checked using a radiometer. During polymerization, light irradiance was maintained at 450 mW/cm^2^ for 30 seconds. After polymerization was completed, all specimens were stored in 37°C distilled water for 24 hours. These procedures in this paragraph were performed as described in previous head.

#### 2.2 Shear bond strength measurement and statistical analysis

Shear bond strengths were measured after 24-hour storage in distilled water. For each of the four groups, specimens were mounted on a universal testing machine (Autograph DCSC-2000, Shimadzu, Kyoto, Japan) and then shear stress was applied at a crosshead speed of 0.5 mm/minute. The edge of loading blade was applied just onon to the interface between luting material and test surface. Data of USC and NUSC groups of each material were statistically compared using the Mann-Whitney rank-sum test.

#### 2.3 Scanning electron microscope (SEM) observation

Before the shear test, a scanning electron microscope (SEM; DS-720, Topcon Corp., Tokyo, Japan) was used to detect and observe morphological changes in the bonding surface due to sandblasting and the presence of aluminum oxide particles with and without ultrasonic cleaning. For each material, the bonding surfaces of polished-only, USC, and NUSC specimens were observed.

After specimens were dried in a desiccator for 24 hours, they were coated with a thin layer of osmium using Neo Osmium Coater (Neoc-ST, Meiwafosis, Tokyo, Japan) before SEM observation at 10 kV.

#### 2.4 Energy dispersive X-ray spectroscopy (EDS) analysis

Before the shear test, energy dispersive X-ray spectroscopy (EDS; Apollo XV, EDAX Inc., Mahwah, NJ, USA) analysis was carried out to determine the elemental compositions of the bonding surfaces of polished-only, USC, and NUSC specimens of both materials. Data were obtained using a SEM (DS-720, Topcon Corp., Tokyo, Japan) with an EDS X-ray detector attached to a take-off angle of 31 degrees. Primary electron beam voltage varied from 10 kV for zirconia ceramic to 15 kV for Co-Cr alloy.

The element distribution maps of zirconia ceramic and Co-Cr alloy specimens subjected to different surface treatments were obtained in the following conditions. The dwell time was 1 msec / 512 X 400 pixels frame, and 300 frames were captured. Semi-quantitative atomic percent maps were calculated by a standardless ZAF correction method.

## Results

### 1 Comparison of adhesive strengths of resin luting material to aluminum oxide and dental restorative materials

Table 1 presents the means and standard deviations of shear bond strength between resin luting material and three dental restorative materials. Mean shear bond strength exhibited by aluminum oxide was significantly higher than those by zirconia ceramic (*P =* 0.021) and Co-Cr alloy (*P =* 0.031). However, there were no statistically significant differences between zirconia ceramic and Co-Cr alloy (*P =* 0.872).

**Table 1:** Means and standard deviations of shear bond strength (MPa) between resin luting material (Panavia F 2.0) and dental restorative materials.

Commercial name	dental restorative materials	Mean	SD
VITA in-Ceram ALUMINA	aluminium oxide block	4.18 ^ab^	(1.77)
LAVA	zirconia ceramic	2.78 ^b^	(1.26)
Cobaltan	Co-Cr alloy	2.88 ^a^	(1.25)

n = 11, Statistical analysis: one way ANOVA and Fisher LSD Method. Mean values represented with same superscript lower case letter shows significant difference

### 2 Effect of alumina particle remnants on adhesive strength between resin luting material and sandblasted surfaces of zirconia ceramic and Co-Cr alloy

Table 2 presents the shear bond strengths of USC and NUSC specimens of zirconia ceramic and Co-Cr alloy. For both zirconia ceramic (P<0.001) and Co-Cr alloy (P = 0.006), NUSC specimens showed significantly higher bond strengths than USC specimens.

**Table 2:** Means and standard deviations of shear bond strength (MPa) of sandblasted surfaces of zirconia ceramic and cobalt-chromium (Co-Cr) alloy with and without ultrasonic cleaning.

Group	Mean	SD
Zirconia ceramic with ultrasonic cleaning	9.67 ^a^	(3.11)
Zirconia ceramic without ultrasonic cleaing	13.11 ^a^	(3.37)
Co-Cr alloy with ultrasonic cleaning	9.80 ^b^	(5.19)
Co-Cr alloy without ultrasonic cleaning	13.60 ^b^	(5.45)

n = 20, Statistical analysis: Mann-Whitney rank Sum test. Mean values represented with same superscript lowercase letters shows significant difference

### 3 SEM observation

**Fig 1:**
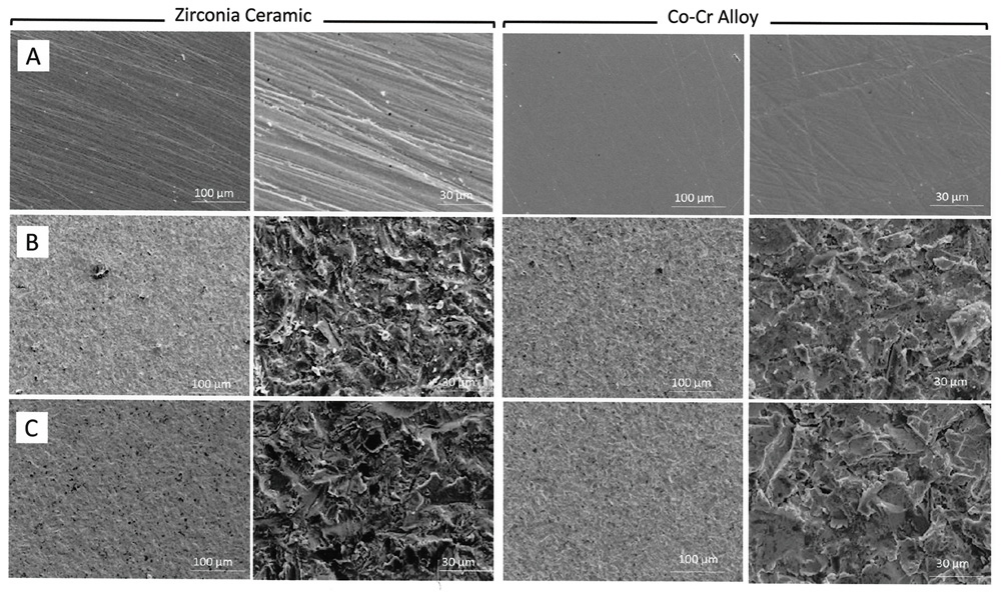
SEM images of the bonding surfaces of zirconia ceramic and cobalt-chromium (Co-Cr) alloy subjected to different surface treatments before shear test. (A) Polish with abrasive paper; (B) Sandblasting; (C) Ultrasonic cleaning after sandblasting.

Fig 1 shows the SEM images of the bonding surfaces subjected to different surface treatments before the shear test. SEM images of the surfaces which were only polished with #1200 silicon carbide abrasive paper showed shallow straight-line grooves. A roughened, irregular surface was obtained after sandblasting for both restorative materials.

For NUSC specimens of zirconia ceramic, abrasive particle remnants were observed on the sandblasted surface. For the other NUSC and USC specimens of both materials, it was difficult to visually discern the abrasive particles from the SEM images.

### 4 EDS analysis

**Fig 2:**
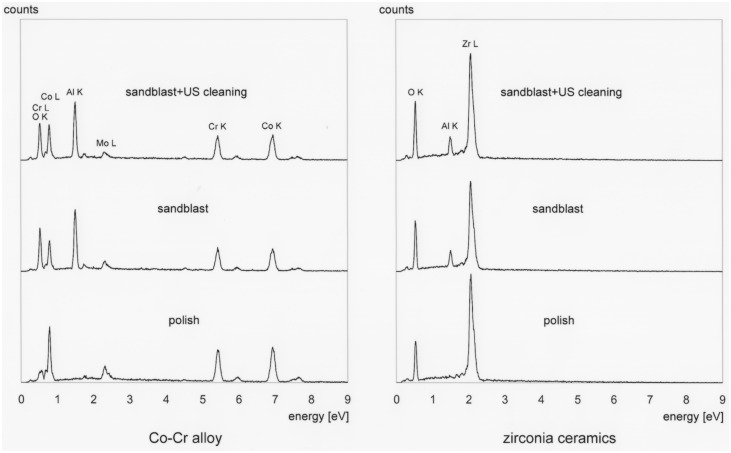
Energy-dispersive X-ray spectroscopy (EDS) spectra of bonding surfaces subjected to different surface treatments.

Fig 2 shows the EDS spectra of the bonding surfaces subjected to different surface treatments. The spectra of NUSC specimens of both materials clearly revealed the presence of aluminum oxide on their bonding surfaces. Even after ultrasonic cleaning, the spectra of USC specimens of both materials still revealed the presence of aluminum oxide.

**Fig 3:**
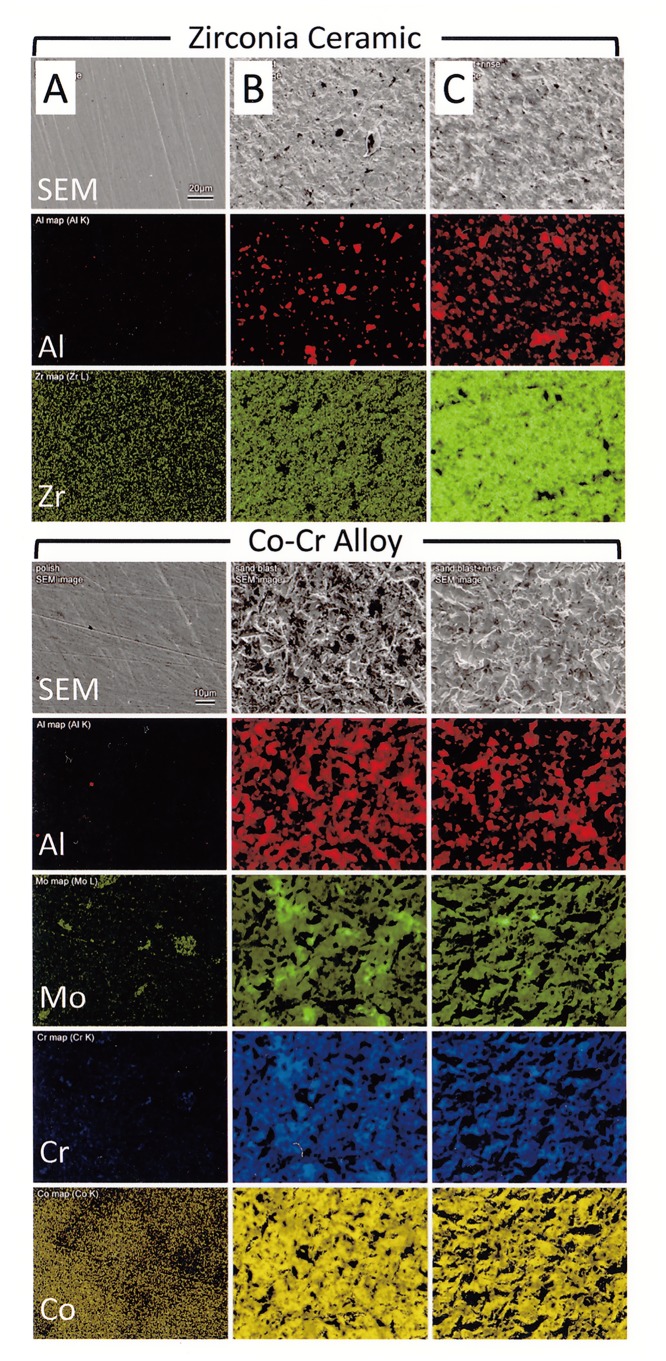
**SEM images (top) and EDS maps showing the element distributions of Al and Zr on zirconia ceramic surfaces:** (A) Polish with abrasive paper; (B) Sandblasting; (C) Ultrasonic cleaning after sandblasting. **SEM images and EDS maps showing the element distributions of Al, Mo, Cr, and Co on Co-Cr alloy surfaces:** (A) Polish with abrasive paper; (B) Sandblasting; (C) Ultrasonic cleaning after sandblasting.

Fig 3 shows the element distribution maps of zirconia ceramic and Co-Cr alloy specimens subjected to different surface treatments. For both zirconia ceramic and Co-Cr alloy, aluminum was still observed even in the USC specimens.

## Discussion

### 1 Bond strength of aluminum oxide versus those of zirconia ceramic and Co-Cr alloy

Bond strength of resin luting material (Panavias F 2.0) to alumina (VITA In-Ceram) was higher than those to zirconia ceramic and Cr-Cr alloy (Table 1). VITA In-Ceram was assumed to the material of blasting particles, because bond strength between luting material ans blasting particle coud not observe. Therefore, sandblasting using aluminum oxide particles might produce increased adhesion efficiency between resin luting material and the aluminum oxide particles blasted onto zirconia ceramic and Co-Cr alloy surfaces, like in the case of tribochemical silica coating. This possibility was further investigated by measuring the bond strengths of sandblasted surfaces with and without ultrasonic cleaning.

### 2 Effect of blasted aluminum oxide particles on bond strength

For both zirconia ceramic and C-Cr alloy, the bond strength of NUSC group was higher than that of USC group (Table 2).

In the SEM image of the NUSC specimen of zirconia ceramic, remnants of blasted aluminum oxide particles could be observed, further bolstering the claim that blasted aluminum oxide particles contributed to enhancing the adhesive strength. However, it was difficult to visually discern the aluminum oxide particles in the rest of the SEM images (Fig 1). Small alumina fragment of particle caused by the blasting pressure might remain on the surface that could not observe in SEM image. This might be due to the limitation of SEM analysis technique.

EDS analysis results clearly revealed the presence of aluminum oxide in NUSC specimens of both restorative materials (Figs 2 and 3). Even after ultrasonic cleaning, EDS results still showed the presence of aluminum oxide on the bonding surfaces. This meant that some degree of blasted aluminum oxide remained in the bonding surface even after it was cleaned with ultrasonic cleaner.

Phase transition of the blasted zirconia ceramic surface occurred during sandblasting [11]. Material volume increased when the tetragonal phase of zirconia transformed to monoclinic phase, that might allow some amount of the more adherent aluminum oxide particles to be firmly trapped and embedded in the zirconia surface, and which were resistant to the cleaning/rinsing action of the ultrasonic cleaner.

Results obtained in this study have shown that ultrasonic cleaning of the bonding surface after sandblasting decreased the adhesive strength of resin luting material, and therefore ultrasonic cleaning of sandblasted surfaces should be avoided. However, there was as yet insufficient data on the amount and magnitude of bond strength of alumina fragment of blasted particles adherent on the bonding surface.

### 3 Other factors of sandblasting that influence bond strength

This study also examined the possibility of blasted aluminum oxide particles providing similar bonding mechanism as tribochemical silica coating. Results obtained suggested that aluminum oxide particles which remained on the bonding surface after sandblasting were effective in increasing bond strength. However, we could not definitively conclude that aluminum oxide particles adherent on zirconia ceramic surface was the only cause of and sole contributing factor to the increased bond strength. Other possible aspects, and hence effects, of sandblasting should be discussed.

Aluminum oxide is an adsorbent for phosphoric acid [12], in that phosphoric acid is chemically adsorbed on aluminum oxide. In this study, the bonding surfaces were coated with aluminum oxide particles after sandblasting, which were an absorbent for phosphoric acid. The resin luting material used, Panavia F 2.0, contained MDP (10-methacryloyloxydecyl dihydrogen phosphate) which is a phosphate monomer. Therefore, the coating of an adsorbent for phosphoric acid was effective in increasing the bond strength between resin luting material and the bonding surfaces.

Sandblasting causes a new surface layer, underlying the top bonding surface, to be exposed. This new surface layer is high in purity and activity [13]. By virtue of its high surface energy, it has a high tendency to attract and combine with other chemical compounds, which then causes its surface energy to decrease [13, 14]. In this study, the pure new surface of zirconia ceramic was highly active and preferentially combined with phosphate group—for zirconia ceramic also has high adsorption capacity for phosphate [15]. For Co-Cr alloy, chromium oxide was instantaneously formed on the new surface upon exposure to the atmosphere [14]. Chromium oxide is highly active and combined with the phosphate group [11].

A highly active surface has high chemical affinity, which also means that it is particularly prone to contamination. Consequently, the new surface created by sandblasting loses its high activity because of contamination. It is highly probable that some contamination of the highly active surface occurs during the ultrasonic cleaning process, which then causes the bonding surface to lose its bonding ability. In this study, stringent contamination control measures were exercised in the handling of all equipment. However, with highly active—and hence highly contaminant-absorbent—bonding surfaces on the one hand, and introduction of contaminants *via* the ultrasonic cleaner or the drying period on the other hand, it was nearly impossible to achieve perfect contamination control. Therefore, it is reasonable to presume that contamination of sandblasted surfaces easily occurs in dental clinics and laboratories when ultrasonic cleaning is used.

### 4 Limitations and conclusion

In this study, increased bond strength was achieved between a phosphate monomer-containing resin luting material and zirconia ceramic and Co-Cr alloy. However, combinations of other luting materials and restorative materials may yield different bond strength results. Moreover in some clinical situations, ultrasonic cleaning is absolutely necessary from the hygiene perspective. These were some of the limitations of the present study, and further investigation is needed to reveal a fuller scope of the unnoticed effects of sandblasting on adhesive strength between resin luting materials and dental restorative materials.

It could be concluded that sandblasting not only roughened the surfaces of restorative materials, but also increased the bond strength between a resin luting material and dental restorative materials. Ultrasonic cleaning of dental restorations after sandblasting should be avoided to retain the improved bond strength between these materials.
